# Sleep and dreaming are for important matters

**DOI:** 10.3389/fpsyg.2013.00474

**Published:** 2013-07-25

**Authors:** L. Perogamvros, T. T. Dang-Vu, M. Desseilles, S. Schwartz

**Affiliations:** ^1^Sleep Laboratory, Division of Neuropsychiatry, Department of Psychiatry, University Hospitals of GenevaGeneva, Switzerland; ^2^Department of Neuroscience, University of GenevaGeneva, Switzerland; ^3^Swiss Center for Affective Sciences, University of GenevaGeneva, Switzerland; ^4^Department of Exercise Science, Center for Studies in Behavioral Neurobiology, Concordia UniversityMontreal, QC, Canada; ^5^Department of Psychology, University of Namur Medical SchoolNamur, Belgium; ^6^Alexian Brother Psychiatry ClinicHenri-Chapelle, Belgium

**Keywords:** sleep, dreaming, emotion, memory, learning, reward system, creativity

## Abstract

Recent studies in sleep and dreaming have described an activation of emotional and reward systems, as well as the processing of internal information during these states. Specifically, increased activity in the amygdala and across mesolimbic dopaminergic regions during REM sleep is likely to promote the consolidation of memory traces with high emotional/motivational value. Moreover, coordinated hippocampal-striatal replay during NREM sleep may contribute to the selective strengthening of memories for important events. In this review, we suggest that, via the activation of emotional/motivational circuits, sleep and dreaming may offer a neurobehavioral substrate for the offline reprocessing of emotions, associative learning, and exploratory behaviors, resulting in improved memory organization, waking emotion regulation, social skills, and creativity. Dysregulation of such motivational/emotional processes due to sleep disturbances (e.g., insomnia, sleep deprivation) would predispose to reward-related disorders, such as mood disorders, increased risk-taking and compulsive behaviors, and may have major health implications, especially in vulnerable populations.

## Introduction

Sleep is a reversible condition of reduced responsiveness usually associated with immobility. All animal species present some form of sleep (or *rest*, which is considered as the sleep equivalent in reptiles, amphibians, fish, and invertebrates), and all need recovery sleep when staying awake longer than usual (i.e., increased sleep pressure) (Cirelli and Tononi, 2008). Sleep contributes to several basic physiological functions pertaining to immunity, hormonal regulation, thermoregulation, and ontogenesis, for example (Morrissey et al., 2004; Van Cauter et al., 2008; Opp, 2009). Conversely, sleep deprivation has deleterious consequences, like increased blood pressure, increased risk for diabetes, obesity, decrease of growth hormones (Van Cauter et al., 2008), and can even be fatal (e.g., in flies and rats) (Rechtschaffen and Bergmann, 2001; Cirelli and Tononi, 2008). Yet, cognitive and emotional disturbances may represent the most noticeable and immediate effects of sleep deprivation. Thus, sleep may serve essential neurological and psychological functions. Reactivation of circuits responsible for memory, emotion, and reward processing during sleep is consistent with this possibility. Indeed, in recent years, neuroimaging and neurophysiological studies have provided accumulating evidence of activated emotional and reward networks during sleep in both humans and animals (Maquet et al., 1996; Braun et al., 1997; Nofzinger et al., 1997; Lena et al., 2005; Dahan et al., 2007; Lansink et al., 2008). These activations seem to be related to the reprocessing and consolidation of memories with a high affective and motivational relevance for the organism (Perogamvros and Schwartz, 2012). Sleep would therefore promote adapted cognitive and emotional responses in the waking state, like performance improvement, emotional balance, and social cognition, among others (as will be discussed in detail below). Furthermore, reward activation in sleep is also supported by the overt expression of reward and emotional behaviors during sleep, as observed in some parasomnias, and by the fact that other sleep disturbances like insomnia and chronic sleep loss may contribute to the development of neuropsychiatric diseases, including mood disorders and addiction. We have proposed that the Reward Activation Model (RAM) may account for these seemingly disparate observations (Perogamvros and Schwartz, 2012).

Below, we ask how dreaming relates to the specific patterns of neural and behavioral activations observed during sleep, in particular whether dreaming may contribute to the offline reprocessing of emotions, associative learning, and exploratory behaviors. We first review existing evidence for the activation of emotional-limbic and reward-related circuits during sleep. We then describe the distinct consequences of such activations during sleep and dreaming for waking functions, including memory, emotions, social skills, and creativity. Because sleep supports essential emotional functions, sleep disturbances have intimate and complex relationships with neuropsychiatric illness, as we also discuss. We conclude by addressing the selectivity of neural and mental processes occurring during sleep, as opposed to those occurring during wakefulness.

## Activation of emotional and reward circuits during sleep and dreaming

Robust evidence for the activation of emotional and reward networks during sleep and dreaming comes from neuroimaging, neurophysiological, and behavioral studies in animals (Lena et al., 2005; Dahan et al., 2007) and humans (Maquet et al., 1996; Braun et al., 1997; Schenck and Mahowald, 2002; Cantero et al., 2003; Desseilles et al., 2011; Perogamvros et al., 2012). Lesion (Solms, 1997, 2000) and pharmacological (Gaillard and Moneme, 1977; Balon, 1996; Pinter et al., 1999; Thompson and Pierce, 1999) studies of dreaming as well as dream content analysis (Nielsen et al., 1991; Merritt et al., 1994; Malcolm-Smith et al., 2012) studies in humans also support such an implication of emotion and reward-related processes in dreams.

### Neuroimaging studies in humans

According to early neuroimaging studies, the distribution of brain activity during sleep is not homogeneous and is characterized by specific activation and deactivation patterns (e.g., Maquet et al., 1996; Braun et al., 1997; Nofzinger et al., 1997). More recent studies using imaging methods with higher temporal and/or spatial resolution (e.g., functional MRI, high-density EEG) reveal more transient changes in brain activity (e.g., Dang-Vu et al., 2008) and in brain connectivity (e.g., Massimini et al., 2005, 2010; Koike et al., 2011) across different sleep stages.

During NREM sleep, decreases in brain activity compared to wakefulness have been consistently found across multiple and distributed brain structures, in agreement with a homeostatic need for brain energy restorative processes. More specifically, decreases of brain perfusion and brain glucose metabolism in the brainstem, thalamus, basal ganglia, basal forebrain and across several cortical areas including the medial prefrontal cortex (mPFC) and precuneus have been demonstrated (Dang-Vu et al., 2010). However, more recent studies have revealed event-related increases of blood-oxygen-level-dependent (BOLD) responses within NREM sleep. In particular, NREM sleep spindles seem to be associated with increased BOLD responses in the lateral and posterior thalamus, as well as in emotion-related regions such as the anterior cingulate cortex (ACC), insula and superior temporal gyrus; in addition, fast spindles are associated with increased activity in the mPFC and hippocampus (HC) (Schabus et al., 2007). Slow waves on the other hand are associated with BOLD increases in the inferior frontal gyrus, brainstem, parahippocampal gyrus, precuneus and posterior cingulate cortex during NREM sleep (Dang-Vu et al., 2008). Finally, one preliminary study (14 subjects) demonstrated bilateral increases in regional glucose metabolism during the transition from waking to NREM in the ventral striatum (VS), anterior cingulate cortex, and extensive regions of the medial temporal lobe, including the amygdala and HC (Nofzinger et al., 2002).

During REM sleep, as compared to wakefulness, a first set of emotion-related regions are activated, including the HC, bilateral amygdala, and ACC (Maquet et al., 1996; Braun et al., 1997; Nofzinger et al., 1997). Amygdala, along with the brainstem, form interactions involved in cardiovascular regulation during sleep (Desseilles et al., 2006) and could reflect responses to intense emotions, in particular fear and anxiety, often experienced in dreams (Schwartz and Maquet, 2002; Smith et al., 2004). Amygdala connections with HC, thalamus, the septal nuclei, mPFC and ACC may also have an important role in strengthening affective value associated to memories (Sterpenich et al., 2007, 2009).

During REM sleep, several structures implicated in executive and attentional functions are significantly deactivated compared to the waking state, including the dorsolateral prefrontal cortex (dlPFC), orbitofrontal cortex (OFC), precuneus, and the inferior parietal cortex (Maquet et al., 1996, 2000, 2005; Braun et al., 1997; Nofzinger et al., 1997). Decreased brain perfusion and glucose metabolism in these regions may cause disorientation, illogical thinking, reduced cognitive control, and impaired working memory in dreaming (Hobson et al., 1998, 2000; Schwartz and Maquet, 2002; Schwartz, 2004; Pace-Schott, 2011).

### Neurophysiological studies

Some key structures of the brain reward circuit, like the ventral tegmental area (VTA) and the nucleus accumbens (NAcc) are also activated during sleep (Solms, 2000; Perogamvros and Schwartz, 2012). In the context of the Reward Activation Model (RAM), we suggest that activation of this network of regions contributes to the reprocessing of memories with a high emotional or motivational relevance during sleep, in coordination with emotion-related circuits (Perogamvros and Schwartz, 2012). For example, during NREM sleep, a spontaneous reactivation (replay) of neuronal firing patterns occurs in VS neurons of rats after a reward searching behavior (Pennartz et al., 2004; Lansink et al., 2008). This off-line replay is induced by HC ripples and may be related to linking a memory trace to a motivational and emotional value during sleep (Lansink et al., 2009; Singer and Frank, 2009; Pennartz et al., 2011). In humans, HC and VS are also activated during NREM sleep (Nofzinger et al., 2002; Peigneux et al., 2004) (see above subsection Neuroimaging Studies). Note that it is unlikely (but still unclear) that the VTA significantly contributes to these processes occurring during NREM sleep, because activity of VTA neurons is relatively low during NREM sleep (Dahan et al., 2007). By contrast, VTA bursting activity is elevated during REM sleep of rats, up to levels observed during reward and punisher anticipation (Carter et al., 2009) and response to stimulus novelty (Bunzeck and Duzel, 2006; Wittmann et al., 2007; Krebs et al., 2011) at wake. Two studies have until now demonstrated that dopaminergic neurons in the VTA of rats express an increased bursting activity during REM sleep, inducing a large synaptic dopamine release in the nucleus accumbens (NAcc) shell (Maloney et al., 2002; Dahan et al., 2007). This activity is significantly higher in REM sleep compared to waking and to NREM and is comparable in intensity and duration to VTA activations during waking behaviors such as feeding, punishment, or sex (Dahan et al., 2007).

Activity in the NAcc is greatest when uncertainty about outcomes is maximal (Cooper and Knutson, 2008). It has been shown that there was an increase in extracellular levels of dopamine in the NAcc during REM sleep of rats (Lena et al., 2005). Other reward-related regions, like the ventromedial PFC (vmPFC) and the ACC, which assign a positive or negative value to future outcomes (Takenouchi et al., 1999; Bush et al., 2002; Haber and Knutson, 2010), are activated during REM sleep in humans (Maquet et al., 1996, 2000). In addition, the HC exhibits a theta rhythm during REM sleep in both animal (Winson, 1972; Popa et al., 2010) and human studies, although human theta activity may be more sporadic and of lower frequency compared to the theta in rodents (Cantero et al., 2003; Clemens et al., 2009). Increased theta oscillatory activity has been found in association with novelty-seeking, exploratory and instinctual behaviors (Panksepp, 1998), which are also overtly present in REM sleep behavior disorder and other parasomnias (Morrison et al., 1995; Jouvet, 1999; Perogamvros et al., 2012). Finally, the orexin neurons in the lateral hypothalamus, related to emotional processing and motivated behaviors (Harris et al., 2005; Schwartz et al., 2008; Ponz et al., 2010a,b; Thompson and Borgland, 2011), have occasional burst discharges during REM sleep of rodents (Mileykovskiy et al., 2005; Takahashi et al., 2008).

**Figure 1:**
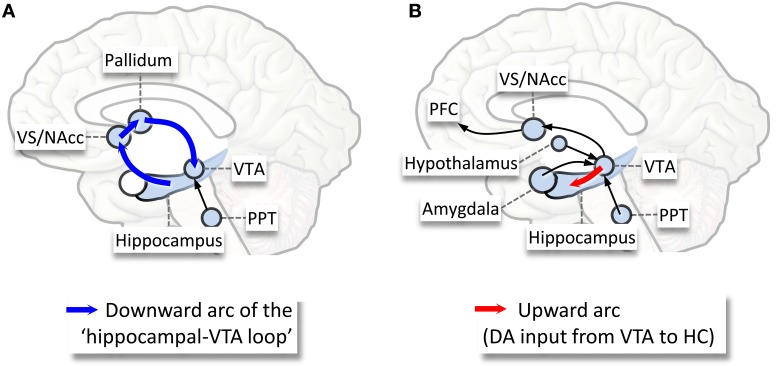
**Schematic illustration of the activation of the mesolimbic-dopaminergic reward system during sleep, and the possible functional interactions within this system, as proposed by the Reward Activation Model (RAM) (Perogamvros and Schwartz, 2012). (A)** During NREM sleep, the activation between hippocampus and VS allows a spontaneous reactivation (replay) of reward-related neuronal firing patterns in the VS, which would involve a transfer of novelty/relevance signal from the hippocampus to the VTA (blue arrows). VTA would be activated during the transition from a NREM episode to REM sleep, with induction of both tonic (hippocampus–VTA projection) and phasic (PPT–VTA) increase of dopamine. Other emotion- and reward-related structures activated during NREM sleep include the amygdala, the ACC and the insula. **(B)** During all REM sleep, increased bursting activity (phasic response) in the VTA may represent stimulus saliency and could fulfill reward-related functions, like acquisition of stimulus-reward associations, novelty-seeking and enhancement of learning procedures. During REM sleep, several VTA projections are activated, including the hippocampus (red arrow), the NAcc, the amygdala, the orexin/hypocretin neurons, the ACC, and the PFC. All these regions have strong anatomical and functional links with the hippocampus and VTA (among others). Abbreviations: ACC, anterior cingulate cortex; PFC, prefrontal cortex; PPT, penduculopontine tegmental nuclei; VS/NAcc, ventral striatum/nucleus accumbens; VTA, ventral tegmental area. Adapted from Perogamvros and Schwartz (2012).

The neurophysiological evidence described above thus suggests that the mesolimbic-dopaminergic (ML-DA) reward and other instinctual exploratory motivational networks (SEEKING system) (Panksepp, 1998) are activated during sleep in mammals (Perogamvros and Schwartz, 2012). Figure 1 provides a plausible schema for the activation and functional interactions within the mesolimbic reward circuitry during NREM and REM sleep. This proposal is also inspired by recent models of hippocampal-VTA functions (Lisman and Grace, 2005).

### Studies of parasomnias

Parasomnias are sleep disorders that involve abnormal behaviors during sleep. Most parasomnias present dissociated sleep features, such as partial arousals during the transitions between wakefulness and NREM sleep (e.g. sleepwalking, confusional arousals, sleep terrors, sleep sex, sleep driving, sleep-related eating disorder, sleep violence), or during REM sleep (e.g., REM sleep behavior disorder, nightmares) (Howell, 2012). REM sleep behavior disorder typically occurs when there is a loss of normal muscle atonia during REM sleep resulting in overt motor behavior during dreaming (Schenck and Mahowald, 2002). It can be caused by adverse reactions to certain drugs or in neurodegenerative disorders such as Parkinson disease, multiple system atrophy, and Lewy Body Dementia. On the other hand, NREM parasomnias are characterized by a state dissociation, in which two states (wake, sleep) occur simultaneously (Bassetti et al., 2000). In addition, it has been proposed that inhibition of supraspinal influence on locomotor centers is an additional condition for NREM parasomnias to occur (Tassinari et al., 2005). While these mechanisms may explain motor disinhibition during the NREM or REM episode, the pathophysiological mechanisms underlying the specific variety of behaviors expressed during parasomnia episodes remain unresolved. In particular, exploratory and instinctual behaviors in humans are very frequently observed in parasomnias: oriented locomotion in sleepwalking, aggression in REM sleep behavior disorder, sexual behaviors in confusional arousals, and feeding, chewing, or swallowing in the sleep-related eating disorder (SRED) (Morrison et al., 1995; Shapiro et al., 2003; Vetrugno et al., 2006; Winkelman, 2006). These complex motor behaviors are most often characterized by negative affect (screaming, crying) (Oudiette et al., 2009), high motivational value (chewing, swallowing, and sexual behaviors), or even compulsive character (SRED, sleep- related smoking disorder) (Provini et al., 2008).

Preliminary data also support a positive correlation between individual daytime reward profile (e.g., novelty seeking, reward sensitivity) and motivational character of behavior during parasomnia episodes (Perogamvros et al., 2012) or in dreams (Johnson, 2001; Colace et al., 2010). The expression of reward networks in the form of overt behavior in sleep would thus require two concomitant conditions: (a) disinhibition of the central pattern generators of the spinal cord, a condition indispensable for parasomnias (Howell, 2012), and (b) elevated daytime exploratory approach, especially toward a certain kind of stimulus. Interestingly, many of the reward-related behaviors observed in parasomnias occur during N2 and N3 sleep stages (Howell, 2012). Moreover, motor and skill consolidation processes take place predominantly during NREM sleep (Walker et al., 2002a; Stickgold and Walker, 2007) and hippocampal ripples, which prevail during this sleep stage (coupled with the sleep spindles) (Clemens et al., 2011), have been implicated in learning and reward processes during sleep (Pennartz et al., 2004; Lansink et al., 2008).

### Lesion, pharmacological and dream content analysis studies

Elevated dopamine level in the ML-DA system during sleep has been suggested to play an important role in the generation of dreams (Solms, 2000). Lesions in the white matter surrounding the frontal horns of the lateral ventricles can cause the cessation of dreaming in humans, without affecting REM sleep (Solms, 1997, 2000). These lesions typically disrupt the ML-DA connections from the VTA to the shell of the NAcc, amygdala, HC, ACC and frontal cortex (insular and medial OFC, medial frontal cortex, vmPFC) (Solms, 2000). This circuit corresponds to the mesolimbic circuits of the SEEKING system, which is a “curiosity-interest-expectancy” command system associated with instinctual appetitive craving states (Panksepp, 1998; Alcaro and Panksepp, 2011). It was thus proposed that the SEEKING system, a psychobehavioral emotional and motivational system subserving approach behaviors and emotional anticipation, is responsible for the generation of dreaming (Solms, 2000; Perogamvros and Schwartz, 2012). It should be noted that, while Panksepp's conceptualization is particularly suitable for addressing the role of the reward system in dreaming, it is compatible with other models of reward system such as those focusing on the reinforcement-learning functions of the reward system (Schultz, 2010a), the reward prediction error (Schultz, 1997, 2002; Redgrave et al., 1999; Schultz and Dickinson, 2000) or the 'incentive salience' (Berridge, 2007). Specifically regarding Berridge's model, “wanting” shares conceptual similarities with Panksepp's SEEKING system, as it corresponds to the organism's motivation, behavioral selection, and switching of attention toward reward-related stimuli and is associated mainly with dopaminergic signaling in mesolimbic structures (Berridge et al., 2009). Supplementary evidence that dreaming may be causally related to mesolimbic activation is sparse and mainly comes from pharmacological studies showing that the administration of dopaminergic agents in humans elicits vivid dreams (Balon, 1996; Pinter et al., 1999; Thompson and Pierce, 1999). On the other hand, administration of D2 antagonists is associated with a reduction in vivid dreaming (Gaillard and Moneme, 1977) and in nightmares (Jakovljevic et al., 2003; David et al., 2004; Lambert, 2006).

Most recent findings show that ‘approach' behaviors in dreams are significantly more prevalent than 'avoidance' behaviors (Malcolm-Smith et al., 2012). Because approach implicates reward-related seeking brain mechanisms, these findings further support the hypothesis that reward-seeking mechanisms are activated during sleep. Importantly, the predominance of approach behaviors is not incompatible with the high prevalence of negative affect in dreams (Nielsen et al., 1991; Merritt et al., 1994; Revonsuo, 2000), because approach/avoidance behaviors and positive/negative emotions are independent psychological entities that engage partially dissociated brain circuits (mesolimbic dopaminergic system and amygdala-limbic system, respectively). For example, if the dreamer approaches an unknown place despite feeling scared, that is an instance of “approach” behavior coinciding with a negative affect (Malcolm-Smith et al., 2012). In any case, both approach and avoidance behaviors share the common feature of salience. Therefore, it seems that emotional, salience and stress networks, subserved by the fear expression (amygdala, ACC), mesolimbic (VTA, NAcc, vmPFC) and extra-hypothalamic (extended amygdala) systems respectively, may collaborate or be in opposition during dreaming, according to the emotion/behavior/cognition characterizing each individual dreamed situation.

The “dopaminergic” theory of dreams, has been criticized by some authors (Hobson and Pace-Schott, 1999; Doricchi and Violani, 2000). Indeed, the influence of dopamine agonists or antagonists on dreaming may be partly mediated by a modulation of REM sleep states (Hobson and Pace-Schott, 1999). Moreover, lesions performed with leucotomy cited in Solms (Solms, 1997) may alter not only dopaminergic, but also cholinergic and noradrenergic efferent and afferent connections (Doricchi and Violani, 2000). Therefore, effects of dopamine on dreaming may be related to its interactions with other neuromodulatory systems, like basal forebrain cholinergic cells that activate cortical and limbic structures (Perry and Piggott, 2000). Besides, dream enhancement can also be observed in patients receiving a noradrenergic beta-receptor blocker (Thompson and Pierce, 1999) or cholinesterase inhibitors (Zadra, 1996). Consequently, interaction of dopaminergic with other neuromodulatory systems must be taken into consideration when studying the neurobiology of dreaming.

## Roles of activation across emotional/motivational networks during sleep

### Off-line memory consolidation

One important function of sleep is memory consolidation, which can be regarded as an open-ended process by which new memories are progressively reorganized and incorporated into pre-existing long-term memory networks (Wang and Morris, 2010). There is accumulating evidence that memory replay and consolidation processes may occur during both REM and NREM sleep in humans (Maquet, 2001; Stickgold, 2005; Diekelmann and Born, 2010; Oudiette and Paller, 2013). Both slow oscillations and sleep spindles during NREM sleep have been reported to be associated with memory (procedural and declarative) consolidation and synaptic plasticity processes (Huber et al., 2004; Rosanova and Ulrich, 2005; Marshall et al., 2006; Bergmann et al., 2008; Diekelmann and Born, 2010; Fogel and Smith, 2011). Besides, neuroimaging studies have demonstrated that both NREM and REM sleep may foster lasting neural changes as well as changes in functional connectivity after perceptual, motor, or emotional learning tasks (Schwartz et al., 2002; Maquet et al., 2003; Sterpenich et al., 2007; Payne and Kensinger, 2010).

On the one hand, activation of the HC and VS during NREM sleep may favor the replay of memory traces comprising contextual, emotional and motivational components (Lansink et al., 2009). Such coordinated reactivation of both HC and VS during NREM sleep provides a possible mechanism for the consolidation of associative memory-reward information (Lansink et al., 2009). In particular, the activation of reward-related neurons in the VS during NREM sleep seems important for selecting memories with a high storage priority (Lansink et al., 2008). Consistent with this hypothesis, declarative memory and skill consolidation observed during sleep is influenced by emotional relevance and motivational biases (Sterpenich et al., 2009; Wilhelm et al., 2011). Further evidence comes from a human study showing that NREM-dependent consolidation of motor skills can be enhanced when it is linked with an anticipation of reward (Fischer and Born, 2009) (but see Tucker et al., 2011). Specifically, NREM-dependent improvement of a motor finger sequence was significantly greater when the sequence was associated with a future monetary reward. Another study demonstrated how such NREM-specific memory processes can be expressed in the form of an overt behavior in sleepwalking (Oudiette et al., 2011).

On the other hand, active memory processing could also explain the reward-related sustained bursting activity of the VTA (Dahan et al., 2007) and the increased dopamine in the NAcc (Lena et al., 2005) during REM sleep. The phasic VTA dopamine signals during REM sleep (Dahan et al., 2007) may favor an off-line replay of recent emotional memory traces during this sleep stage (Walker and Van Der Helm, 2009). These memories could then also serve as salient stimuli for VTA because the way that ML-DA neurons code for reward is strongly influenced by the individual's needs and relevant memories (e.g., emotional events, current concerns) (Schultz, 2010b). At the same time, memories could also engage the ML-DA system whenever they represent novel or unanticipated stimuli (Schultz and Dickinson, 2000), such as when they are activated in the absence of associated contextual information and/or of cognitive control from dlPFC during REM sleep (Fosse et al., 2003; Schwartz, 2003; Pace-Schott, 2011). Importantly, memory and reward processes may form reciprocal relations, as ML-DA activation is necessary for memory formation and learning (Adcock et al., 2006; Shohamy and Adcock, 2010) (Figure 1B).

REM sleep was found to promote neuronal reorganization of emotional memory traces involving the progressive transfer of memories along a hippocampal-amygdalo-cortical route. More specifically, studies by Ribeiro and colleagues (Ribeiro et al., 2002; Ribeiro and Nicolelis, 2004) demonstrated an upregulation of the gene zif-268 [related to the expression of long-term potentiation, LTP (Richardson et al., 1992)] in the amygdala, entorhinal, auditory, somatosensory and motor cortices during REM sleep in rats. Further support for the role of REM sleep in LTP processes comes from studies showing that REM sleep deprivation negatively affects LTP in the visual cortex (Shaffery et al., 2002) and in the dorsal HC (Ravassard et al., 2009) of rats. In humans, functional MRI studies have found that REM-dependent enhanced functional connectivity between the amygdala and the mPFC subserve long-lasting consolidation of emotional memories (Sterpenich et al., 2009; Payne and Kensinger, 2011). Yet, despite robust limbic activation during REM sleep, the studies above suggest that all stages of sleep contribute to the consolidation of relevant memories (i.e., with an emotional or motivational value) involving substantial long-term reorganization within cerebral networks.

### Emotional regulation

Both SWS and REM sleep have been linked to emotion regulation processes (Lara-Carrasco et al., 2009; Gujar et al., 2011a; Pace-Schott et al., 2011; Talamini et al., 2013). Several studies suggest that REM sleep may have a “depressiogenic” effect, and could possibly be deleterious for the emotional well-being. This idea comes from the observation that awakenings from an REM episode are characterized by an increased depressive score in the Hamilton Rating Scale for Depression (McNamara et al., 2010) and an enhanced aversive reactivity toward familiar affective stimuli (Wagner et al., 2002; Lara-Carrasco et al., 2009) [but see also (Gujar et al., 2011a; Van Der Helm et al., 2011)]. In addition, an increase in REM density and a reduced REM latency usually precede clinical depression, suggesting that REM sleep, which is under circadian and homeostatic control (Wurts and Edgar, 2000), may constitute a biomarker for depressive disease (Giles et al., 1998; Gottesmann and Gottesman, 2007).

In light of the recent findings on the consequences of total sleep deprivation and REM sleep deprivation on emotional and reward functions at the behavioral (Killgore et al., 2006; Banks and Dinges, 2007), neurobiological (Venkatraman et al., 2007; Yoo et al., 2007; Gujar et al., 2011b; Menz et al., 2012) and clinical (Riemann et al., 2002; Salvadore et al., 2010) levels, we propose here that REM sleep cannot be reduced to a depressiogenic sleep stage, but may instead have a crucial role in the maintenance of the integrity of emotional and reward networks. Indeed, perturbation of these networks with total or partial sleep deprivation leads to reduced disappointment in response to losses, together with decreased activity in the insular cortex (Venkatraman et al., 2007), which is thought to process the emotional significance of a stimulus, including the somatic affective response and awareness (Ernst and Paulus, 2005). Moreover, sleep deprivation caused attenuated conflict sensitivity for trials yielding high decision conflict (relatively large amounts associated with relatively low probability of winning) in a risky choice task, paralleled by a downregulation of conflict-related signals in the SN/VTA (Menz et al., 2012). A failure of top-down cortical control from the mPFC on the amygdala (Yoo et al., 2007), and an amplified reactivity of reward networks in response to positive emotional stimuli (Gujar et al., 2011b) have been also described as consequences of sleep deprivation. It has been also suggested that REM sleep may decrease the emotional reactivity to aversive or stressful experiences (Gujar et al., 2011a; Van Der Helm et al., 2011) (but see Pace-Schott et al., 2011; Talamini et al., 2013). Furthermore, changes in mood after sleep deprivation share several phenomenological similarities with hypomania, and the risk of hypomanic switch is elevated during and after sleep deprivation (Wehr et al., 1982; Colombo et al., 1999). Interestingly, antidepressants that do not suppress REM sleep (e.g., bupropion) (Nofzinger et al., 1995; Ott et al., 2004), have the least risk to induce a hypomanic switch in a depressed person (Leverich et al., 2006). This latter observation is consistent with the proposal that REM suppression is one of the main mechanisms by which most antidepressants and sleep deprivation induce hypomanic/manic symptoms (Salvadore et al., 2010). A subsensitization of presynaptic dopamine receptors (Tufik et al., 1987) and an increased noradrenergic tone (Payne et al., 2002) with REM sleep deprivation (compared with rested sleep) may be implicated in this effect. In sum, these results indicate that REM sleep fosters adapted emotional responses during waking life (Walker and Van Der Helm, 2009) by contributing to overnight emotional and reward brain functions.

The sustained activation of emotional and reward networks during dreams may also serve an emotion regulation function. Evidence in favor of this claim comes from the demonstration that mood in REM dreams improves over the course of a night sleep for subjects with pre-sleep depressed mood (Cartwright et al., 1998). This improvement may putatively be due to experiencing diverse emotional stimuli during dreaming; the exposure to feared stimuli (objects, situations, thoughts, memories, and physical sensations) in a totally safe context during dreaming may thus resemble desensitization therapy (Perlis and Nielsen, 1993; Levin and Nielsen, 2007). Furthermore, sleep was found to promote the retention and generalization of extinction learning, which could potentially be helpful in generalizing therapeutic extinction learning in exposure therapy (Pace-Schott et al., 2009, 2012b). Experiencing negative emotions during dreaming may thus function as a threat simulation system, which ultimately affords adapted emotional responses during waking life (Revonsuo, 2000). Nightmares would by contrast reflect the failure of fear memory extinction, in the presence of temporary (e.g., daily concerns) or more persistent (e.g., trauma) increases in affect load (Nielsen and Levin, 2007). Different REM dream characteristics have been found to relate to distinct facets of daytime affective functioning, such as suicide probability (i.e., frequency of nightmares) (Agargun et al., 2007), protection against depression after divorce (i.e., incorporation of marital status issues) (Cartwright et al., 1984), and response to antidepressant treatment (i.e., reduction of negatively toned dreams) (Schredl et al., 2009).

Extending the Threat Simulation Theory (TST) according to which dreaming allows an offline simulation of threatening events that promotes efficient threat-avoidance skills during wakefulness (Revonsuo, 2000), the Reward Activation Model (RAM) supports that dreaming (and offline processes during sleep) exposes the dreamer to memory and emotional content with a high storage priority, for example relating to instinctual behaviors or drives (such as feeding, mating, fighting, fleeing, etc.), as well as to novel internally-generated stimuli (e.g., original combinations of elements from memory) (Perogamvros and Schwartz, 2012). Based on robust neurophysiological evidence of reward activation during sleep, the RAM thus implies that dreams do not only relate to known past events but also to an unexpected, novel or probabilistic future (see also Perogamvros and Schwartz, 2012; Pace-Schott, 2013). Further supporting this hypothesis, dream content analysis has shown that although past and current waking concerns are common in dreams (Schredl and Hofmann, 2003; Cartwright et al., 2006), dreams are usually novel constructions and rarely reproductions of past events (Fosse et al., 2003; Schwartz, 2003).

While mental activity during REM and NREM sleep vary across the night sleep, with NREM dreams becoming longer, less thought-like and more dream-like and bizarre, thus resembling REM dreams in the morning (Fosse et al., 2004; Wamsley et al., 2007a), dream content analyses report generally higher emotional intensity in REM than in NREM dreams (Smith et al., 2004; McNamara et al., 2005; Wamsley et al., 2007a), consistent with the robust activation of limbic structures during REM sleep. On the other hand, dreamer-initiated friendliness is frequent in NREM dreams, while aggressive social interactions may be more characteristic of REM reports (McNamara et al., 2005). Dream data thus confirm that emotional and motivational processes may be active during both REM and NREM sleep stages, in line with the present proposal (section Activation of Emotional and Reward Circuits During Sleep and Dreaming). However, they also suggest that some types of emotional experience may prevail during specific sleep stages, with more intense negative emotions and aggressiveness possibly predominating in REM dreams.

### Theory of mind in dreams

The reprocessing and consolidation of memories with a high affective or motivational relevance for the organism may prioritize both social cognition and self-representation processes during dreaming. Accordingly, it has been proposed that our ability to attribute mental states to self and others (i.e., theory of mind) may be at least partly preserved in dreams (Pace-Schott, 2001, 2007; Kahn and Hobson, 2005; McNamara et al., 2005). Envy, embarrassment, attachment, sexual attraction, shame, pride, etc. are often reported in dreams, thus supporting the idea that the dreamer is exposed to both his/her own emotions and the expression of emotions in other dream characters. Moreover, social interactions as well as attribution of mental states to dream characters appear to be controlled by NREM and REM mechanisms: NREM dreaming would predominantly simulate friendly interactions, self-related information, and actual waking life events, whereas REM dreams contain comparatively more aggressive social interactions (McNamara et al., 2005) and less integration between self-referential and social cognitive reasoning with autobiographical memory (Pace-Schott, 2013). This dissociation fits the more general finding that the expression of instinctual emotions and behaviors (e.g. fear and aggressiveness) is more prevalent in REM than NREM dreams (Smith et al., 2004; Wamsley et al., 2007b), which in turn is consistent with substantially larger activation of the amygdala and other limbic regions in REM relative to NREM sleep (Vandekerckhove and Cluydts, 2010).

Pace-Schott has recently proposed that the activation of the brain's “default network” during sleep may help explain the expression of social skills and affects during NREM and REM sleep (Pace-Schott, 2007, 2013) (see also Domhoff, 2011). The default-mode network encompasses a set of connected brain regions that are thought to be typically activated when individuals are engaged in internally-focused tasks including spontaneous cognition, daydreaming, autobiographical memory retrieval, imagination, and introspection (Buckner et al., 2008). Two main default-network subsystems have been identified: the dorsomedial prefrontal subsystem (dorsomedial prefrontal cortex, lateral temporal cortex, temporoparietal junction and temporal pole), which is selectively activated during self-reflection and attribution of mental states to others, and the medial temporal subsystem (hippocampus-parahippocampus, retrospenial cortex and vmPFC), which is activated during memory retrieval and prospective memory (Buckner et al., 2008; Andrews-Hanna, 2012). While correlations among brain regions of the default-mode network largely persist during N1 and N2 stages of NREM sleep (Laufs et al., 2007; Horovitz et al., 2008; Larson-Prior et al., 2009), there is evidence for reduced frontal connectivity during SWS (Horovitz et al., 2009; Samann et al., 2011) and reduced connectivity within the dorsomedial prefrontal subsystem during REM sleep (Koike et al., 2011). In addition, mPFC and most of the medial temporal lobe subsystem seem activated during REM sleep (Pace-Schott, 2013). These temporal, stage-dependent changes in the connectivity of components of the default-network may explain the distinct roles of NREM and REM stages in the expression of self-reflection, social skills and the “mentalizing” function of sleep. Whether this constitutes a mechanism by which dreams facilitates the resolution of social and emotional conflicts remains to be addressed in future studies (Desseilles et al., 2011).

### Creativity

There are innumerous anecdotal accounts suggesting that sleep enhances creativity and problem solving in artists and scientists. It is said that Robert Louis Stevenson came up with the plot of Strange Case of Dr Jekyll and Mr Hyde during a dream, and that Mary Shelley's Frankenstein was also inspired by a dream at Lord Byron's villa. Paul McCartney purportedly discovered the tune for the song “Yesterday” in a dream and was inspired to write “Yellow Submarine” after hypnagogic auditory hallucinations. Otto Loewi (1873–1961), a German-born physiologist, dreamed of the experiment that ultimately allowed him to prove chemical synaptic transmission, and was later awarded the Nobel Prize. In one of the first experimental studies addressing the relation between sleep and creativity (Home, 1988), participants either underwent 32 h of sleep deprivation or slept normally. The sleep-deprived participants scored low on measures of cognitive flexibility and originality, suggesting that sleep deprivation impairs divergent thinking. More recently, Walker et al. (2002b) demonstrated that subjects awoken after REM sleep had a 32% advantage on an anagram solving task, compared with the number of correct responses after NREM awakenings. In a similar study, (Stickgold et al., 1999) used a semantic priming task and elegantly demonstrated that subjects awoken from REM sleep showed greater priming by weak primes (than by both unrelated primes and strong primes), consistent with a hyperassociative state of the sleeping mind, as also observed in REM dreams. More recently, Cai et al. (2009) showed that compared with quiet rest and non-REM sleep, REM sleep enhances the integration of initially unassociated information resulting in more creative problem solving. In addition, sleep can also facilitate insightful behavior because it involves a restructuration of new memory representations (Wagner et al., 2004; Pace-Schott et al., 2012a).

Activation of the dopaminergic system during sleep likely underlies some of these findings. Indeed, an important function of the dopaminergic system is to favor creativity, including associative thinking, innovative insights, cognitive flexibility and divergent thinking (Akbari Chermahini and Hommel, 2010). Further evidence comes from Parkinson's disease patients under dopaminergic treatment, who may develop increased artistic drive and productivity (Inzelberg, 2013). Moreover, spontaneous creative activity, like in jazz improvisation, has been linked to absence of control from structures that typically mediate conscious volitional control of ongoing performance, like the dlPFC, and simultaneous activation of internally motivated, stimulus-independent behaviors, subserved by the mPFC (Limb and Braun, 2008). Interestingly, dlPFC is deactivated and the mPFC is activated during REM sleep (Maquet et al., 1996), supporting the specific potential role of this sleep stage in human creativity.

## Can sleep disturbances precipitate neuropsychiatric disease?

In the previous section (section Roles of Activation across Emotional/Motivational Networks during Sleep), we showed that sleep and dreaming may have an impact on various aspects of waking cognitive functions (memory consolidation, emotional regulation, self and others representations, and creativity). Yet, any claim about causal relations between sleep and these functions would require further research and considerations. Indeed, physiological parameters other than those related to specific sleep stages, such as circadian processes (circadian phase), homeostatic processes (duration of prior wakefulness) (Boivin et al., 1997), or others, could be responsible for such modulations. It is true that even a minimal misalignment between circadian phase and sleep phase can deteriorate mood (Danilenko et al., 2003), potentially explaining mood-related problems (e.g., irritability) in jet-lag or shift work (Kolla and Auger, 2011), and severity of unipolar depression (Hasler et al., 2010). In addition, mood deteriorates with increased duration of prior wakefulness (Boivin et al., 1997). Thus, we could as well hypothesize that insomnia (i.e., the subjective perception of inadequate sleep) or sleep loss (an objective measure of sleep reduction) does not affect daytime functioning. Below, we provide cases against this statement.

### Sleep disturbances and mood disorders

Sleep deprivation is an objectively measured decrement in sleep, which often has an immediate antidepressant effect in depressed patients (Barbini et al., 1998; Giedke and Schwarzler, 2002). Usually this effect is short-lived, with 50–80% of the responders relapsing after recovery sleep (Giedke and Schwarzler, 2002). Several hypotheses have been formulated to explain this antidepressant effect, including dopaminergic (Ebert and Berger, 1998), noradrenergic (Payne et al., 2002), circadian (Wehr and Wirz-Justice, 1981), and homeostatic (Endo et al., 1997) mechanisms. On the other hand, insomnia is a subjective perception of inadequate sleep that represents an independent major risk of subsequent onset of major depression (Riemann and Voderholzer, 2003; Johnson et al., 2006; Buysse et al., 2008; Roane and Taylor, 2008). Hypomania and mania have also been causally linked to reduced need for sleep (Wehr et al., 1987; Wehr, 1991; Plante and Winkelman, 2008). Indeed, a decrease in sleep may trigger a shift toward hypomania/mania in bipolar patients, whereas an increase in sleep can lead to a shift toward depression (Bauer et al., 2006). In addition, sleep disturbances are the most common prodrome of mania and the sixth most common prodrome of depression (Jackson et al., 2003).

Which neurophysiological mechanisms may explain such functional links between sleep disturbances and mood disorders? As depression is also considered as a risk factor for insomnia (Ohayon and Roth, 2003), it has been proposed that the reciprocal links between mood disorders/sleep disturbances form a closed loop (Harvey, 2008). Circadian (Wirz-Justice, 2003; Roybal et al., 2007; Li et al., 2013) and neuroendocrine (Schmider et al., 1995; Spiegel et al., 1999; Holsboer, 2000) mechanisms may partially, but not totally subserve a direct causality between sleep disturbances and mood disorders. Activation of emotional and reward networks during sleep as proposed here may offer a supplementary and necessary mechanism. Indeed, conditions of chronically disrupted sleep, like in insomnia or chronic sleep deprivation, are known to produce reward-related and memory-related deficits, such as deficits in working memory, episodic memory and executive functioning (Van Dongen et al., 2003; Fortier-Brochu et al., 2012), decreased global emotional intelligence, empathy toward others and quality of interpersonal relationships (Killgore et al., 2008), increased aggressiveness (Kamphuis et al., 2012), and negative mood states (Dinges et al., 1997; Zohar et al., 2005). Impaired overnight consolidation of declarative memory is also observed in patients with primary insomnia (Backhaus et al., 2006). In addition, chronic insomnia is characterized by structural abnormalities of reward-related and memory-related structures such as the OFC (Altena et al., 2010), the HC (Riemann et al., 2007), and the ACC (Plante et al., 2012). Cumulative functional deficits in the integrity of emotional and reward networks because of sleep deprivation (as presented in the subsection Emotional Regulation) may also add to chronic structural abnormalities. Similar reward-related deficits, such as decreases in the capacity to seek out rewards, in decision-making (aboulia), and in the ability to experience pleasure (anhedonia), are also characteristics of depression (Der-Avakian and Markou, 2012), and depict a dysfunction across mesolimbic and non-mesolimbic reward networks (Tremblay et al., 2005; Pizzagalli et al., 2009; Blood et al., 2010). In addition, reward dysfunction in hypomania/mania, at both the neurophysiological (Blumberg et al., 2000; Rubinsztein et al., 2001; Abler et al., 2008) and the phenomenological (increased risk taking, impulsivity) (Mason et al., 2012) levels, is also observed.

Taking into consideration such deficits in both insomnia and mood disorders, we propose that sleep disturbances may be causally related to mood disorders, notably by altering reward processing in a way that would precipitate the reward-deficient symptomatology found in depression/mania. Future studies are needed to clarify if specific characteristics of sleep restrictions (in microstructure, macrostructure, duration or quality of sleep) predict specific manic or depressive symptoms. It should be also noted that, as already mentioned, unipolar depression or bipolar disorder cannot be reduced only to a consequence of sleep disturbances and reward-related deficits, as their pathophysiology is characterized by complex interactions between genetic, biological, psychological and social determinants.

### Sleep disturbances and compulsivity

Compulsive behaviors like drug addictions are characterized by an inability to reduce the occurrence of an approach behavior toward a primary or secondary reward (e.g., food, drug), and by a negative emotional state when the access to the reward is precluded. It is now well-established that compulsive behaviors may relate to a dysregulation of the hedonic (OFC, insula), emotional (amygdala), and motivational (VTA, NAcc) components of the reward networks (Koob, 2009; Koob and Volkow, 2010). Recent findings suggest that sleep disturbances like insomnia, sleep deprivation and circadian misalignment may developmentally precede and predict early onset of alcohol, cigarette and marijuana use in adolescents and young adults (Roane and Taylor, 2008; Wong et al., 2009; Hasler and Clark, 2013). A disruption of brain reward networks, as well as ineffective emotion regulation processes due to sleep disturbances, could explain the compensatory drug and food seeking in sleep deprived animals (Puhl et al., 2009) and humans (Benedict et al., 2012; Telzer et al., 2013). Disturbances of neuroendocrine (Van Cauter et al., 2008) mechanisms may also be implicated in these interactions. Conversely, sustained cocaine abstinence was shown to cause symptoms of chronic insomnia that may increase the probability for future relapse (Morgan et al., 2006). Future longitudinal studies would be useful to further substantiate and clarify these links between sleep disturbances and compulsive behaviors.

## Sleep and dreaming: what the waking state cannot offer

We have provided evidence supporting that both REM and NREM sleep may offer a neurobehavioral substrate for offline reprocessing of emotion or associative learning (section Roles of Activation across Emotional/Motivational Networks during Sleep). In particular, we suggest that by critically supporting learning and memory processes, sleep may influence the development and maintenance of waking consciousness (Hobson, 2009; Hobson and Friston, 2012). In addition, sleep disturbances may trigger waking state neuropsychiatric disorders (section Can Sleep Disturbances Precipitate Neuropsychiatric Disease?). Importantly, sleep seems to serve primordial neural and cognitive functions that cannot be provided by wakefulness (Cirelli and Tononi, 2008). Specifically, the neural traces coding for newly acquired information are reactivated during subsequent periods of both SWS and/or REM sleep, thus leading to enhancement of memory performance in waking (Maquet et al., 2000; Maquet, 2001; Stickgold, 2005; Rasch et al., 2007; Rudoy et al., 2009; Diekelmann and Born, 2010; Oudiette and Paller, 2013). Reactivation of organized sequences of behavior in humans are also supported by the report that trained motor sequences are replayed in the form of overt behavior in patients with parasomnia (Oudiette et al., 2011). It has been shown that memories are destabilized during waking memory reactivation (being thus vulnerable to interference), but stabilized by memory reactivation occurring during SWS (Diekelmann et al., 2011). Moreover, recent experimental evidence also demonstrated that, after training on a virtual navigation task, reactivation of task-related mentation in dreams improved performance, and significantly more than when task-related thoughts occurred during wakefulness (Wamsley et al., 2010). Together, these findings support the existence of learning and memory processes that are specific to the sleep and dream states, thus making them important for waking performance.

Distinct sleep stages and dream states (associated with NREM or REM sleep) may also differ in their contribution to off-line reprocessing of emotional and reward information. NREM sleep may be more specialized in linking memory traces with motivational values (Pennartz et al., 2004; Lansink et al., 2009), whereas REM sleep is responsible for emotional memory consolidation, and synaptic consolidation (Wagner et al., 2001; Sterpenich et al., 2009; Diekelmann and Born, 2010; Popa et al., 2010; Payne and Kensinger, 2011). Whether REM and NREM dreams reflect distinct facets of sleep-dependent memory consolidation is unclear, and this is partly due to the fact that studies combining dream content analysis with an assessment of emotion and/or memory functions are scarce (Cartwright et al., 1998; Agargun et al., 2007; Wamsley et al., 2010). Hence, whether and how conscious experience in dreams causally contributes to the functions discussed in this article (i.e., memory consolidation, emotional regulation, associative learning, and creativity) remains hypothetical. Moreover, due to the specific brain states (i.e., REM and NREM sleep) in which dreaming occurs and the reduced sensory and contextual anchoring of the dream elements, memory for dreams is often poor as compared to everyday life memories (e.g., Schwartz and Maquet, 2002). While it is tempting to relate some aspects of dreaming to cognitive and emotional functions, as we do in this review and as others have done in the past (Threat Simulation Theory (Revonsuo, 2000), protoconsciousness theory (Hobson, 2009), default-mode activation theory (Pace-Schott, 2007; Domhoff, 2011), reward activation theory (Solms, 2000; Perogamvros and Schwartz, 2012), we need to acknowledge that data is still lacking in this domain to support any strong conclusions. Methodological improvements in the integration of self-report data with neuroimaging data represent a promising avenue for future developments in the science of dreaming (Horikawa et al., 2013; Perogamvros, 2013).

## Conclusions

There is robust evidence supporting the activation of limbic, paralimbic and reward structures during NREM and REM sleep. These activations subserve important high-level cognitive functions, such as memory consolidation, emotional regulation processes, theory of mind, and creativity. Thus, waking consciousness is at least partly shaped by neural and mental processes occurring during sleep and dreaming. The contribution of sleep and dreaming to some specific functions (e.g., those pertaining to memory consolidation and creativity) appears to be unique, dissociated from similar processes occurring during wakefulness, and associated to specific sleep stages and possibly dream states. Some other functions, such as emotional regulation and social cognition, may benefit from dreaming, as this can provide an offline, virtual and safe environment, in which the dreamer can be exposed to an important load of rewarding or aversive stimuli. More empirical studies are needed to better characterize the potential roles of dreaming. Conversely sleep disturbances, such as insomnia and sleep deprivation, result in emotional distress and reward-related dysfunction, as frequently found in mood disorders and addiction. Future longitudinal studies could be useful to further substantiate and clarify these links. By presenting converging lines of evidence supporting the role of sleep in learning and emotion regulation, one of our goals would also be to encourage the development of preventive measures for populations, like children and psychiatric patients, who may be particularly vulnerable to the deleterious consequences of sleep loss.

### Conflict of interest statement

The authors declare that the research was conducted in the absence of any commercial or financial relationships that could be construed as a potential conflict of interest.

## Abbreviations

ACC: anterior cingulate cortex
dlPFC: dorsolateral prefrontal cortex
HC: hippocampus
ML-DA: mesolimbic dopaminergic system
mPFC: medial prefrontal cortex
N1: N2, N3, stages N1, N2, and N3 of NREM sleep
NAcc: nucleus accumbens
NREM sleep: non-rapid-eye movement sleep
OFC: orbitofrontal cortex
PFC: prefrontal cortex
PPT: pendunculopontine tegmental nucleus
RAM: Reward Activation Model
REM sleep: rapid-eye movement sleep
SRED: sleep-related eating disorder
SWS: slow-wave sleep
TST: threat simulation theory
vmPFC: ventromedial prefrontal cortex
VS: ventral striatum
VTA: ventral tegmental area.
